# Unraveling the potential of CD8, CD68, and VISTA as diagnostic and prognostic markers in patients with pancreatic ductal adenocarcinoma

**DOI:** 10.3389/fimmu.2024.1283364

**Published:** 2024-01-30

**Authors:** Fereshteh Rezagholizadeh, Fatemeh Tajik, Morteza Talebi, Seyed Reza Taha, Mahdieh Shariat Zadeh, Pooya Farhangnia, Hamideh Sadat Hosseini, Aram Nazari, Shabnam Mollazadeh Ghomi, Seyede Mahtab Kamrani Mousavi, Niloofar Haeri Moghaddam, Hossein Khorramdelazad, Mohammad Taghi Joghataei, Elahe Safari

**Affiliations:** ^1^ Cellular and Molecular Research Center, Iran University of Medical Sciences, Tehran, Iran; ^2^ Department of Molecular Medicine, Faculty of Advanced Technologies in Medicine, Iran University of Medical Sciences, Tehran, Iran; ^3^ Department of Immunology, School of Medicine, Iran University of Medical Sciences, Tehran, Iran; ^4^ Oncopathology Research Center, Iran University of Medical Sciences, Tehran, Iran; ^5^ Department of Medical Genetics and Molecular Biology, Faculty of Medicine, Iran University of Medical Sciences, Tehran, Iran; ^6^ Human and Animal Cell Bank, Iranian Biological Resource Center (IBRC), Tehran, Iran; ^7^ Immunology Board for Transplantation and Cell-Based Therapeutics (ImmunoTACT), Universal Scientific Education and Research Network (USERN), Tehran, Iran; ^8^ Immunology Research Center, Institute of Immunology and Infectious Diseases, Iran University of Medical Sciences, Tehran, Iran; ^9^ Department of Pathology, School of Medicine, Iran University of Medical Sciences, Tehran, Iran; ^10^ Department of Medical Nanotechnology, Faculty of Advanced Technologies in Medicine, Iran University of Medical Sciences, Tehran, Iran; ^11^ Department of Immunology, School of Medicine, Rafsanjan University of Medical Sciences, Rafsanjan, Iran

**Keywords:** VISTA, PDAC, CD68, pancreatic ductal adenocarcinoma, prognosis, biomarker, CD8

## Abstract

**Introduction:**

Pancreatic cancer is a truculent disease with limited treatment options and a grim prognosis. Immunotherapy has shown promise in treating various types of cancer, but its effectiveness in pancreatic cancer has been lacking. As a result, it is crucial to identify markers associated with immunological pathways in order to improve the treatment outcomes for this deadly cancer. The purpose of this study was to investigate the diagnostic and prognostic significance of three markers, CD8, CD68, and VISTA, in pancreatic ductal adenocarcinoma (PDAC), the most common subtype of pancreatic cancer.

**Methods:**

We analyzed gene expression data from Gene Expression Omnibus (GEO) database using bioinformatics tools. We also utilized the STRING online tool and Funrich software to study the protein-protein interactions and transcription factors associated with CD8, CD68, and VISTA. In addition, tissue microarray (TMA) and immunohistochemistry (IHC) staining were performed on 228 samples of PDAC tissue and 10 samples of normal pancreatic tissue to assess the expression levels of the markers. We then correlated these expression levels with the clinicopathological characteristics of the patients and evaluated their survival rates.

**Results:**

The analysis of the GEO data revealed slightly elevated levels of VISTA in PDAC samples compared to normal tissues. However, there was a significant increase in CD68 expression and a notable reduction in CD8A expression in pancreatic cancer. Further investigation identified potential protein-protein interactions and transcription factors associated with these markers. The IHC staining of PDAC tissue samples showed an increased expression of VISTA, CD68, and CD8A in pancreatic cancer tissues. Moreover, we found correlations between the expression levels of these markers and certain clinicopathological features of the patients. Additionally, the survival analysis revealed that high expression of CD8 was associated with better disease-specific survival and progression-free survival in PDAC patients.

**Conclusion:**

These findings highlight the potential of CD8, CD68, and VISTA as diagnostic and prognostic indicators in PDAC.

## 1 Introduction

Pancreatic cancer is extremely truculent, the third leading cause of malignancy-associated mortalities in both genders combined in the United States, predicted to become the second-most deadly by 2030 and has the lowest 5-year survival rate of 11% among all cancers (1, 2). The incidence of pancreatic cancer is rising rapidly, by about 1.1% annually across the world (3). Pancreatic cancer is highly metastatic and has a low response to various treatments. Considering that the effectiveness of surgery and adjuvant therapy relies on the early diagnosis of pancreatic cancer, it is necessary to move towards the development of early diagnosis approaches. As a result, this can bring hope for a better treatment prognosis and longer survival. However, the disease is usually detected at an advanced stage when the tumor is unresectable due to the lack of early diagnostic markers and imperceptible symptoms at early stages (4, 5). Considering all these problems, functional diagnostic and prognostic biomarkers as well as novel therapeutic agents are needed to ameliorate patients’ survival rates (6).

Recently, cancer immunotherapy has widely attracted attention, and many attempts have been made to explore new curative strategies. Cancer immunotherapy utilizes the host immune system, especially CD4^+^ and CD8^+^ T cells, to attack tumor cells with different approaches like immune checkpoint inhibitors (ICIs), immunomodulators, and cancer vaccines (7, 8). Immune checkpoints are hijacked by tumors to reduce T-cell immune responses and evade immune surveillance (9). Despite the groundbreaking success of immunotherapy with antibodies against immune checkpoints such as cytotoxic T-lymphocyte-associated protein 4 (CTLA-4), programmed cell death protein 1 (PD-1), and programmed death-ligand 1 (PD-L1) in multiple solid malignancies (10–13), pancreatic cancer remains treatment refractory (14–16). Studies carried out on the effectiveness of ICI treatments involving the use of anti-PD-1 or anti-CTLA-4 antibodies in pancreatic cancer, both alone and in combination, have revealed unsatisfactory overall response rates of 0% and 3%, respectively (17–19). The reason is possibly due to a unique pancreatic tumor microenvironment (TME), which is characterized by abundant stromal content enriched with FAP^+^ cancer-associated fibroblasts, poor vasculature, and immunosuppressive cells such as regulatory T cells, CD68^+^ M2-like tumor-associated macrophages (TAMs), and myeloid-derived suppressor cells (MDSCs) (20, 21). In addition, some studies have demonstrated that CD8^+^ T cells are poorly infiltrated in pancreatic TME (22, 23). However, reports from other publications have shown high infiltration and activity of CD8^+^ T cells in the TME (24, 25). The other possible reason for the ineffectiveness of immunotherapy in pancreatic cancer is the presence of many highly expressed negative immunoregulatory checkpoints in the TME, like T-cell immunoglobulin and mucin domain-3 (TIM-3), T cell immunoreceptor with immunoglobulin and ITIM domain (TIGIT) (26), lymphocyte activation gene-3 (LAG-3), and V-domain Ig suppressor of T cell activation (VISTA) (27). Thus, targeting different and novel antigenic molecules is needed to overcome pancreatic cancer resistance.

VISTA is a B7 family negative immune checkpoint regulator encoded by the *C10orf54* gene (28). Although VISTA and PD-L1 have some structural similarities, their functional pathways are distinct (29). VISTA is predominantly expressed on TME-infiltrating myeloid cells (i.e., MDSCs and CD68^+^ TAMs), CD4^+^ and CD8^+^ T cells, and tumor cells (30–32). However, the expression pattern of VISTA in pancreatic cancer is not completely understood. Moreover, the correlation between VISTA and tumor-infiltrating immune cells is still unclear. Finally, the efficacy of targeted therapy against VISTA remains uninvestigated.

In the current study, we aimed to evaluate the diagnostic and prognostic value of CD8, CD68, and VISTA in pancreatic ductal adenocarcinoma (PDAC), a subtype of pancreatic cancer that accounts for the majority of pancreatic cancer cases. We performed immunohistochemistry (IHC) on several PDAC tissue microarrays (TMAs). This allowed us to accurately quantify the expression levels of these proteins in PDAC tissues and assess their potential as diagnostic and prognostic markers. We also utilized gene expression data to further investigate the potential of these markers as diagnostic and prognostic indicators in PDAC. Our findings can potentially inform the development of more accurate diagnostic tools for PDAC. Overall, our study sheds light on the important roles of CD8, CD68, and VISTA in the progression and prognosis of PDAC and highlights the importance of further research in this area.

## 2 Materials and methods

### 2.1 Gene expression omnibus database

An *in silico* investigation was conducted using the GEO database to determine the expression of CD8A, CD68, and VISTA (C10orf54) in PDAC compared to adjacent normal tissues. The GSE183795, GSE28735, and GSE62452 datasets, encompassing 139, 45, and 69 PDAC and 101, 45, and 61 corresponding adjacent non-cancerous tissues, respectively, were obtained. GEO2R, available at http://www.ncbi.nlm.nih.gov/geo/geo2r/, was utilized to identify differentially expressed genes between the tumor and adjacent non-cancerous pancreatic samples.

### 2.2 Protein-protein interaction (PPI) network and transcription factors identification

The PPI network was created using the online database STRING (version 10.5; http://string-db.org/), with a parameter of medium confidence >0.4 for interactions. Subsequently, the FunRich software was utilized to identify the upstream transcription factors of CD8A, CD68, and VISTA as well as their associated genes. Ultimately, the protein-protein interaction network between the aforementioned transcription factors and the genes CD8A, CD68, and VISTA was constructed via STRING. The interactions were visualized using Cytoscape software (version 3.6.1; http://www.cytoscape.org/).

### 2.3 Pathway enrichment analysis

To investigate the genes that interact with CD8A, CD68, and VISTA, as well as the mentioned transcription factors (TFs), a biological pathway enrichment analysis was conducted using the FunRich tool (http://www.funrich.org) against a human FunRich background database.

### 2.4 Patients’ characteristics and specimens

In this study, 228 samples of formalin-fixed, paraffin-embedded (FFPE) PDAC tissue were collected from patients who underwent surgery at Imam Khomeini University-based hospital in Tehran, Iran, between 2010 and 2020. None of the patients had previously undergone chemotherapy or radiotherapy. Demographic and clinicopathological data, such as gender, age, tumor size, grade, pTNM stage, tumor site, margin involvement, perineural invasion, lymphovascular invasion, lymph node metastasis, macroscopic tumor extension, tumor recurrence, and distant metastasis were obtained from medical records and analyzed. pTNM classification was used to assess the stage of PDAC. Additionally, 10 normal (non-tumor) tissues adjacent to PDAC were considered to compare the protein expression level of VISTA, CD68, and CD8 in normal and tumoral tissues. Disease-specific survival (DSS) and progression-free survival (PFS) were measured by calculating the time between pancreatectomy and death due to pancreatic cancer, and the time between the primary surgery and the most recent follow-up visit without any signs of cancer recurrence or metastasis, respectively. The study was conducted with ethical approval (Code: IR.IUMS.FMD.REC.1399.161) obtained from the Research Ethics Committee of the Iran University of Medical Sciences.

### 2.5 Tissue microarray (TMA) construction

Pancreatic tissue TMAs were constructed. In brief, H&E-stained slides of all specimens were evaluated to determine the most suitable regions of the tumor and normal cells in various parts of the tissue samples. Then, selected areas of each donor block were punched out with a core of 1 mm diameter and precisely assembled into new recipient blocks using TMA equipment (Galileo CK3500 TMA, ISENET, Milan, Italy). Each block contained three copies of different regions from one tumor sample and was scored separately to resolve the tumor heterogeneity issue. Afterwards, 4-µm-thick TMA slides were provided by cutting sections of completed array blocks, which were then transferred to adhesive-coated slides. The mean score of three cores was calculated from each tissue specimen as the final score.

### 2.6 Immunohistochemistry (IHC) staining

The expression of VISTA, CD8, and CD68 immune markers was immunohistochemically investigated on all prepared TMA slides on immune cells. After deparaffinization of sections at 60°C for 40 min and rehydration through xylene followed by a graded ethyl alcohol series, 3% hydrogen peroxide (H2O2) for 20 min was applied at room temperature as an endogenous peroxidase inhibitor. Next, the tissue sections were conducted in three wash steps in Tris-Buffered Saline (TBS), and then the slides for 10 min were immersed in citrate buffer (pH = 6.0) for CD68 and VISTA and Tris-EDTA buffer (pH = 9) for CD8 by autoclaving to carry out antigen retrieval. Subsequently, the tissue slides were individually incubated overnight with primary antibodies against VISTA (rabbit monoclonal antibody, Cat. No. 64953, Cell Signaling Technology, Inc., Danvers, Massachusetts, USA; 1:200 dilution), CD8 (rabbit monoclonal antibody, clone CD8/4391R, Cat. No. RM0409RTU7, Medaysis, Livermore, California, USA; 1:100 dilution), and CD68 (mouse monoclonal antibody, clone KP1, Cat. No. MC0084RTU7, Medaysis, Livermore, California, USA; 1:100 dilution) at 4 °C. To reduce non-specific binding, rabbit immunoglobulin G (Invitrogen, Thermo Fisher Scientific, Waltham, Massachusetts, USA) was used as an isotype control at concentrations similar to those of primary antibodies. Moreover, tonsil tissue for CD8 and CD68 and kidney tissue for VISTA were used as positive controls. Following rinsing three times in Tris-buffered saline, sections were incubated with a secondary antibody (MedaView™ Two-step Polymer-HRP Anti-Mouse & Rabbit System, Cat. No. DP0221, Medaysis, Livermore, California, USA). The chromogenic reaction was visualized by immersing the sections in 3, 3′-diaminobenzidine (Dako, Denmark) for 3 min, and then hematoxylin was used as a nuclear counterstain. Eventually, sections were dehydrated through serial dilutions of ethyl alcohol, cleared in xylene, and finally mounted for evaluation.

### 2.7 Evaluation of immunostaining and scoring system

Samples stained for CD68, CD8, and VISTA were assessed in a blinded manner by two expert pathologists (A.Z. and S.M.) through a semi-quantitative scoring system called H-score. Furthermore, in cases of discrepancies, the final H-score was determined by reaching a consensus between two pathologists. Moreover, Cohen’s kappa coefficient was used to assess inter-rater variability and the level of agreement among raters. The H-score (ranging from 0 to 300) was used as the overall score for each case and was calculated by multiplying the results of two separate scoring systems: the percentage of positive cells (0% to 100%) and the intensity of staining. Immunostaining intensity was scored visually as 0 (absent), 1 (weak), 2 (moderate), and 3 (strong). This study considered the median H-score a setpoint to determine high or low CD68, CD8, and VISTA expression.

### 2.8 Statistical analysis

Statistical analysis of the data generated from GEO was conducted using GraphPad 6 Prism software. To assess the differences in CD8A, CD68, and VISTA expression between groups, unpaired t-tests, Wilcoxon matched-pairs signed-rank tests, and Mann-Whitney tests were utilized. All data were represented as mean ± standard deviation (SD), and a p-value of less than 0.05 was considered statistically significant. All the obtained data were analyzed using the SPSS software version 25.0 (SPSS, Inc., IBM Corp, USA). For the exhibition of categorical information, N (%) and mean with SD and median with quartile (Q1, Q3.) were used for numeric information. Pearson’s χ2 test (Pearson’s chi-squared test) was used to examine the significance of the association between the expression of protein markers and clinicopathological features. Kruskal-Wallis and Mann-Whitney U tests were utilized for pairwise comparisons between the study groups. Moreover, the Kaplan-Meier analysis was used to generate the DSS and PFS curves and to compare the survival outcomes between high- and low-expression groups, a log-rank test with a 95% confidence interval (CI) was carried out. The Cox proportional hazards regression model was applied to determine which variables affected DSS or PFS. Throughout the analysis, *P*<0.05 was considered as the threshold for statistical significance. Moreover, we employed the Benjamini-Hochberg procedure to carefully adjust all p-values (33).

## 3 Results

### 3.1 Analysis of GEO data reveals elevated levels of CD68 and slight alteration of VISTA in PDAC

**Figure 1:**
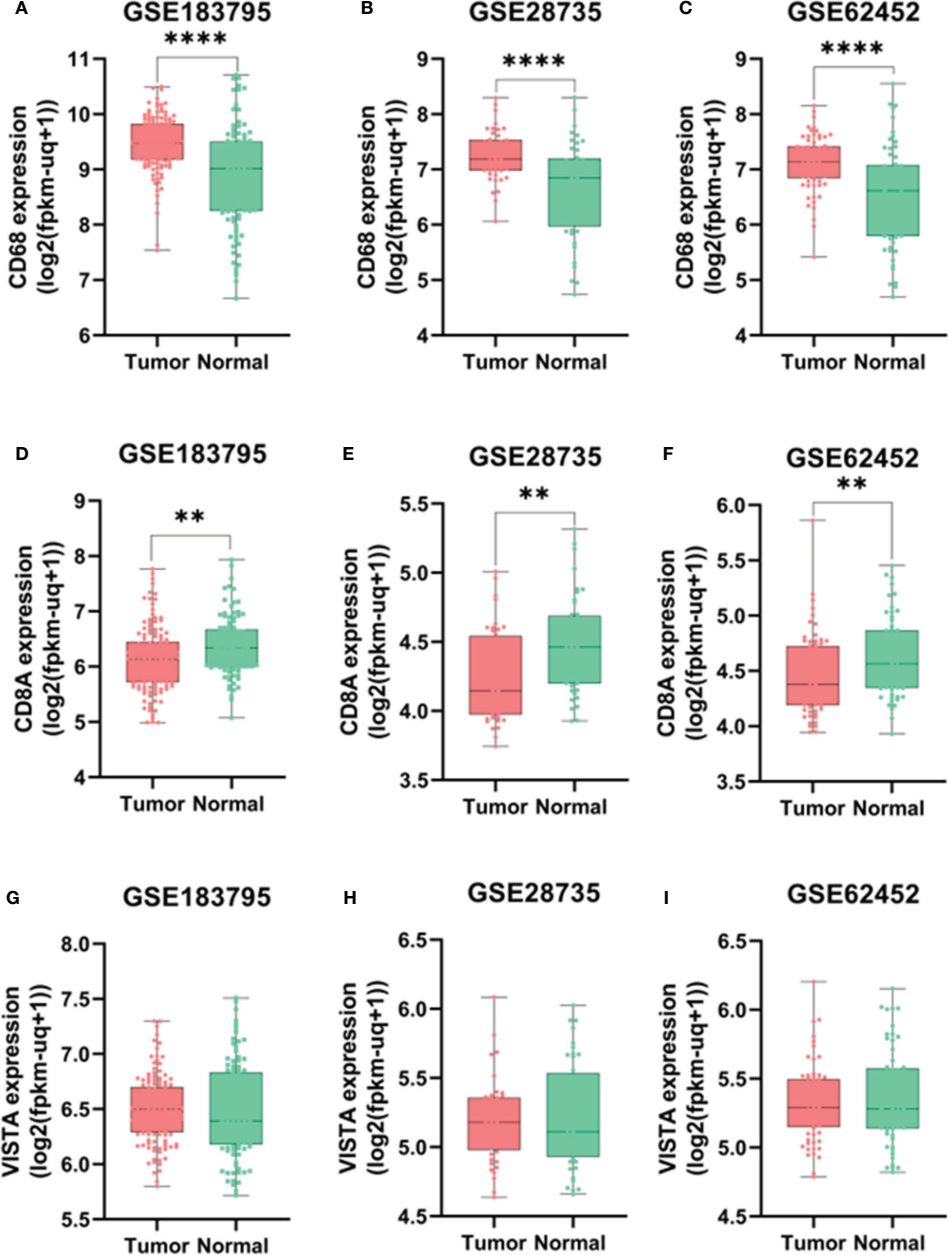
Expression levels of CD68 according to GSE183795 **(A)**, GSE28735 **(B)**, and GSE62452 **(C)** datasets analysis. Expression levels of CD8A according to GSE183795 **(D)**, GSE28735 **(E)**, and GSE62452 **(F)** datasets analysis. Expression levels of VISTA according to GSE183795 **(G)**, GSE28735 **(H)**, and GSE62452 **(I)** datasets analysis. **: P<0.01, ****: P<0.0001.

We conducted an analysis of microarray datasets from the GEO to examine the expression levels of CD8A, CD68, and VISTA in PDAC samples compared to adjacent normal samples. Our analysis revealed that the expression levels of CD68 were significantly higher in PDAC samples compared to adjacent normal samples, as shown in **Figures 1A–C**, with a p-value of less than 0.0001. Conversely, CD8A had significantly lower levels (*P*=0.0013, *P*=0.0018, and *P*=0.0085) in PDAC samples compared to normal samples, as indicated by the three datasets (**Figures 1D–F**). However, the expression level of VISTA showed no significant statistical difference (**Figures 1G–I**). All in all, these findings suggest the potential involvement of immune regulators in the development of PDAC.

### 3.2 PPI network

**Figure 2:**
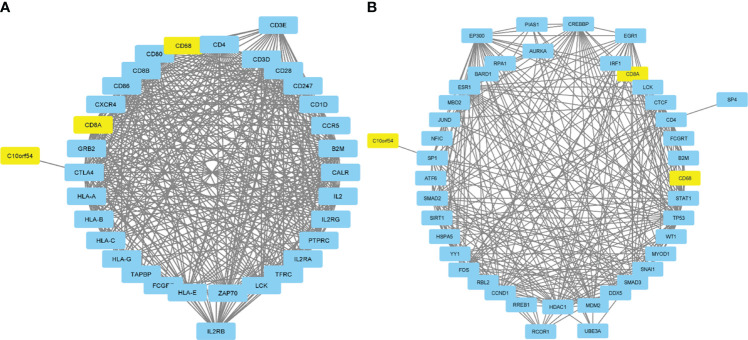
Protein-protein interactions between CD8A, CD68, and VISTA and key players in the regulation of immunological processes **(A)**. Transcription factors that act on the upstream of the mentioned network and their PPI network with CD8A, CD68, VISTA, and related genes **(B)**.

**Figure 3:**
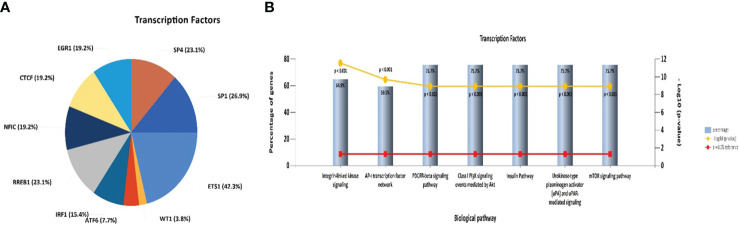
Pie chart of transcription factors that act on the upstream of the mentioned network in **Figure 2A** **(A)**. Association of the mentioned genes in **Figure 2B** with the relevant signaling pathways **(B)**.

An investigation was conducted into potential protein-protein interactions between CD8A, CD68, and VISTA and other proteins. As depicted in **Figure 2A**, a network composed of key players in the regulation of immunological processes was constructed. This network displays the interaction of CD8A, CD68, and VISTA genes with other significant genes, including B2M, CD4, CD80, IL2, CTLA-4, CD86, CD28, and PTPRC. Notably, VISTA is highly connected to CTLA-4, suggesting their involvement in the destruction of cancer cells by T cells. Subsequently, we predicted the transcription factors that act on the upstream of this network (**Figure 3A**) and plotted their PPI network with CD8A, CD68, and VISTA (**Figure 2B**). The analysis of this network reveals that CD8A, CD68, and VISTA can be significantly influenced by critical proteins and transcription factors such as TP53, EP300, ESR1, CCND1, CREBBP, FOS, HDAC1, SMAD3, SMAD2, and STAT1, thereby having a role in cancer tumorigenesis. **Figure 3B** demonstrates the involvement of these genes in the relevant signaling pathways, emphasizing their role in the initiation and progression of cancer.

### 3.3 Patients’ characteristics

In this study, among 228 FFPE tissue specimens, VISTA, CD68, and CD8 were expressed at different intensities in 148, 140, and 124 samples, respectively. The age and tumor sizes were categorized into two groups based on the median. Tumor cells were classified as well, moderately, or poorly differentiated based on their histological grade. All samples were also divided into 4 stages according to the pTNM classification for pancreatic cancer (34). All clinicopathological characteristics for samples with VISTA, CD68, and CD8 expression are summarized in **Table 1**.

**Table 1:** Tumor clinicopathological characteristic of patients with pancreatic ductal adenocarcinoma samples based on VISTA, CD68, and CD8 expression.

Patients and tumor characteristics	Samples with VISTA expression	Samples with CD68 expression	Samples with CD8 expression
**Number of samples**	148	140	124
Age			
**Median Age, years (Range)** ≤ Median age > Median age	59 (12-85) 74 (50.0) 74 (50.0)	60 (19-85) 70 (50.0) 70 (50.0)	59 (12-85) 65 (52.4) 59 (47.6)
sex			
Male Female	81 (54.7) 67 (45.3)	71 (50.7) 69 (49.3)	60 (48.4) 64 (51.6)
Tumor size			
**Median Tumor size (cm) (Range)** ≤ Median > Median	3 (0.3-16.5) 74 (51.0) 71 (49.0)	3 (0.4-10) 77 (55.7) 61 (44.3)	3.3 (0.5-16.5) 61 (50.0) 61 (50.0)
Histological grade			
Well-differentiated Moderate differentiated Poor differentiated	58 (44.9) 59 (45.7) 12 (9.4)	55 (44.3) 58 (46.7) 11 (9.0)	52 (45.2) 55 (47.8) 8 (7.0)
TNM stage			
I II III IV	29 (25.0) 68 (58.6) 15 (12.9) 4 (3.5)	45 (36.2) 58 (46.7) 14 (11.2) 7 (5.9)	33 (31.4) 55 (52.3) 13 (12.3) 4 (4.0)
Margin involvement			
Yes No	31 (25.2) 92 (74.8)	30 (23.4) 98 (76.6)	30 (27.2) 80 (72.8)
Perineural invasion			
Present Absent	83 (66.9) 41 (33.1)	77 (61.1) 49 (38.9)	77 (71.2) 31 (28.8)
Lymphovascular invasion			
Present Absent	61 (54.9) 50 (45.1)	56 (52.8) 50 (47.2)	52 (52.5) 47 (47.5)
Lymph node (LN) metastasis			
Present Absent	63 (52.9) 56 (47.1)	64 (50.7) 62 (49.3)	55 (51.8) 51 (48.2)
Macroscopic tumor extension			
Yes No	91 (71.0) 37 (29.0)	94 (77.0) 28 (23.0)	83 (74.7) 28 (25.3)
Tumor recurrence			
Yes No	13 (9.2) 128 (90.8)	20 (14.7) 116 (85.3)	12 (10.0) 107 (90.0)
Distant metastasis			
Yes No	60 (42.5) 81 (57.5)	59 (43.3) 77 (56.7)	57 (47.8) 62 (52.2)

### 3.4 Expression levels of VISTA, CD68, and CD8 in PDAC compared with adjacent normal samples

**Figure 4:**
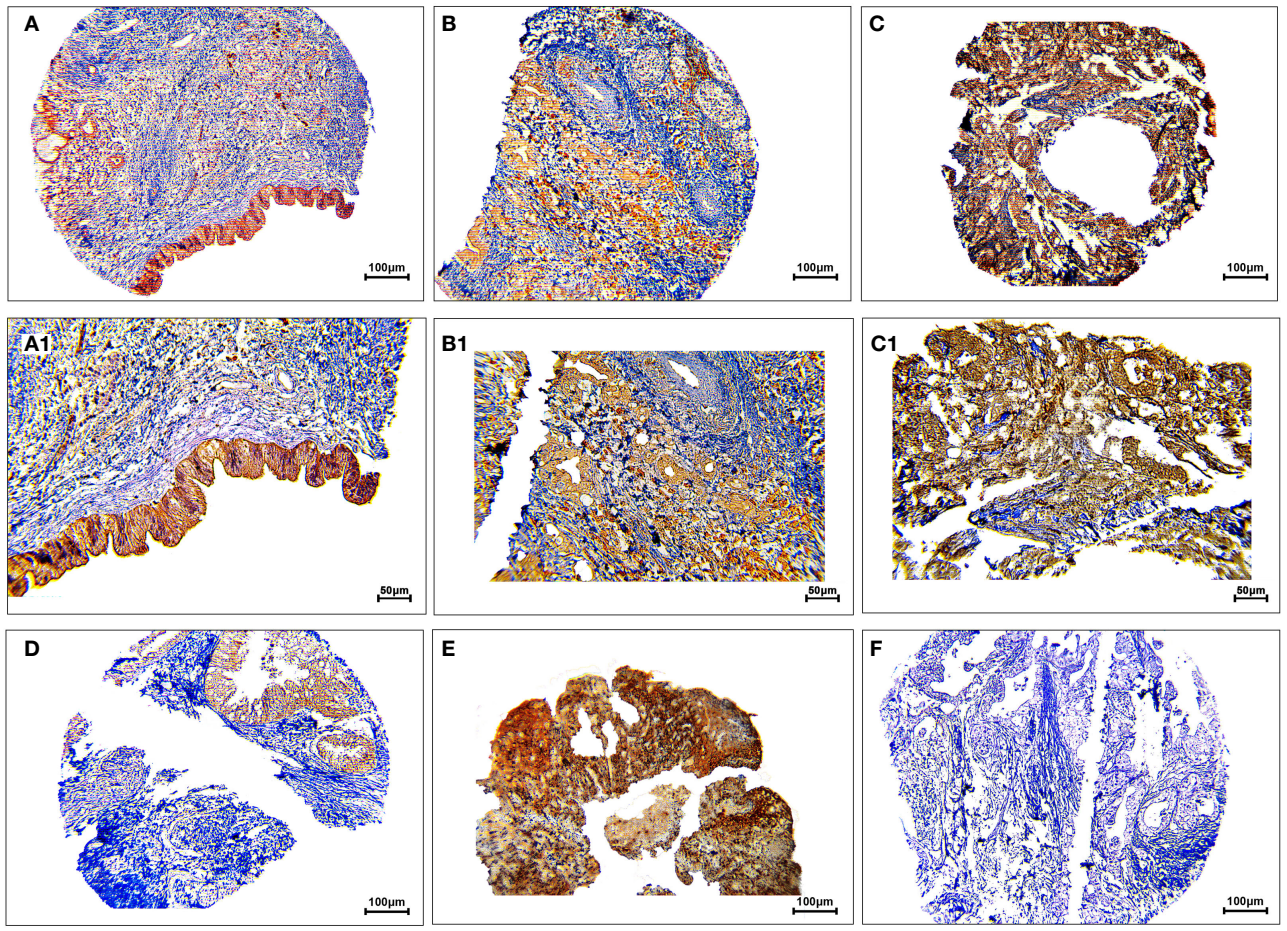
Staining cytoplasmic pattern of CD8 protein expression in pancreatic ductal adenocarcinoma. Representative images illustrate low-intensity expression **(A, A1)**, moderate-intensity expression **(B, B1)**, and high-intensity expression **(C, C1)**. CD8 expression in adjacent non-tumoral tissue **(D)**. CD8 expression in tonsil tissue as a positive control **(E)**. Isotype control **(F)**. Images were taken at 100× **(A–C)** and 200× **(A1–C1)** magnifications.

**Figure 5:**
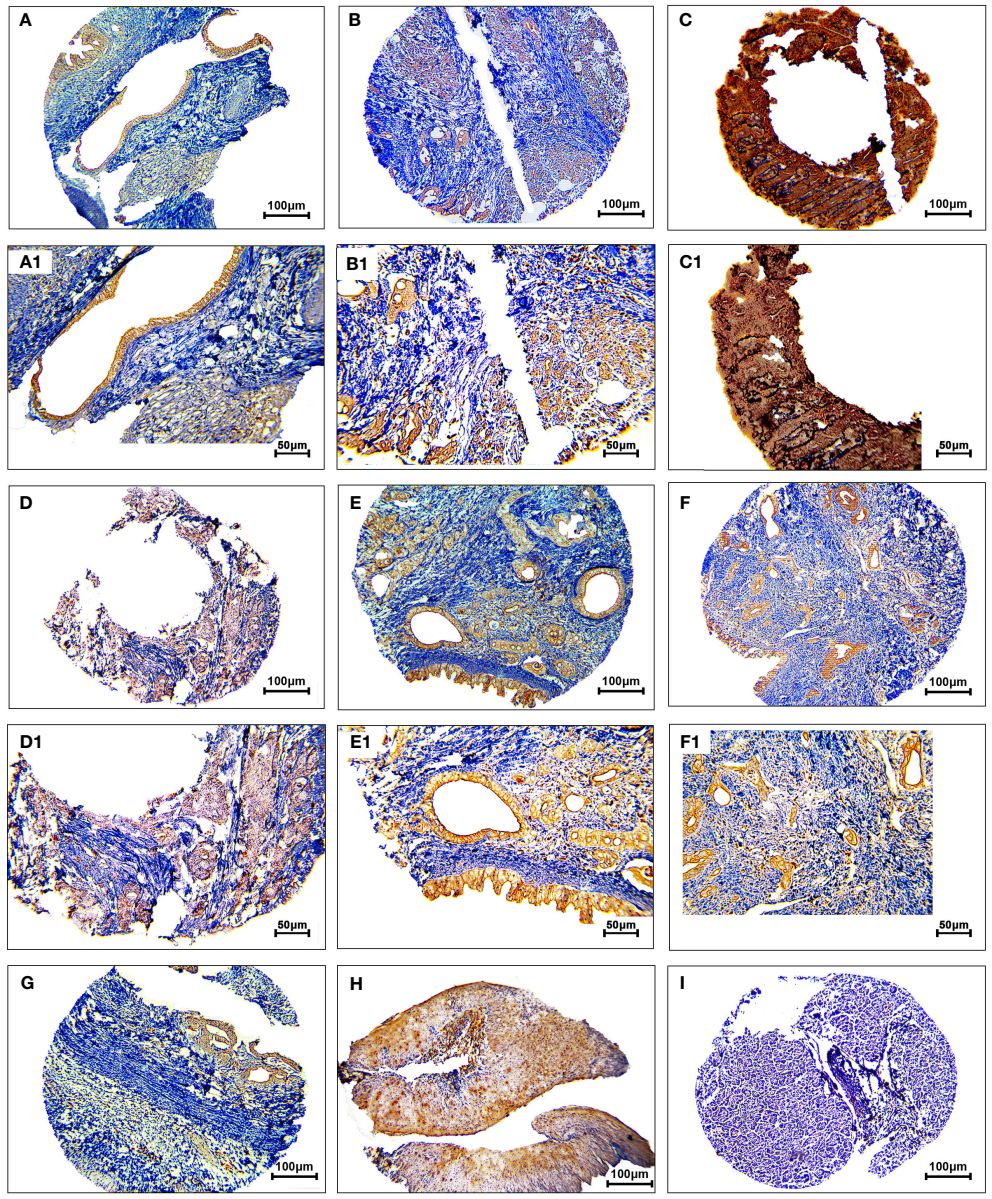
Staining cytoplasmic and membranous pattern of CD68 protein expression in pancreatic ductal adenocarcinoma. Representative images illustrate low-intensity cytoplasmic expression **(A, A1)**, moderate-intensity cytoplasmic expression **(B, B1)**, high-intensity cytoplasmic expression **(C, C1)**, low-intensity membranous expression **(D, D1)**, moderate-intensity membranous expression **(E, E1)**, and high-intensity membranous expression **(F, F1)**. CD68 expression in adjacent non-tumoral tissue **(G)**. CD68 expression in tonsil tissue as a positive control **(H)**. Isotype control **(I)**. Images were taken at 100 × **(A–F)** and 200× **(A1–F1)** magnification.

**Figure 6:**
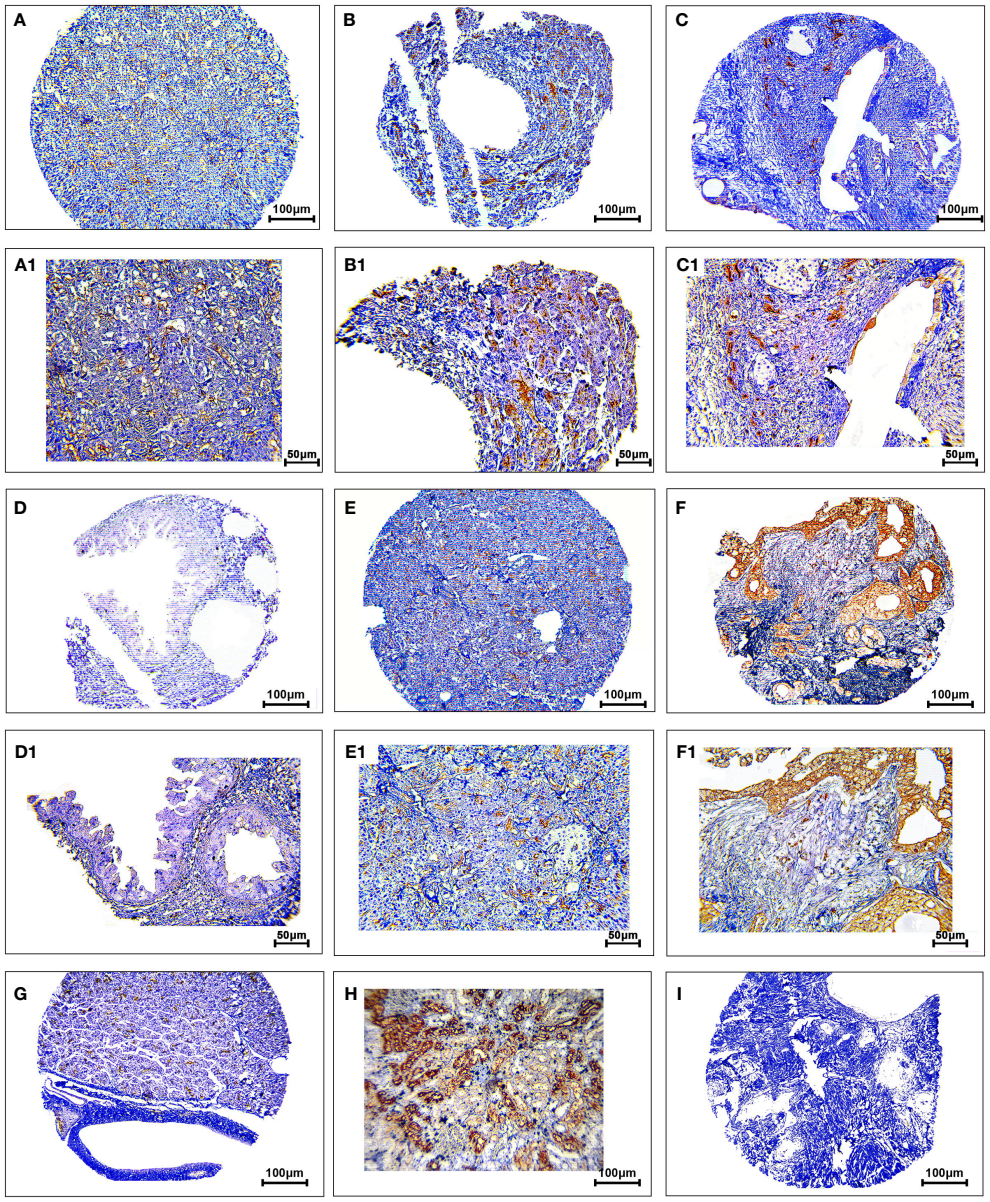
Staining cytoplasmic and membranous pattern of VISTA protein expression in pancreatic ductal adenocarcinoma. Representative images illustrate low-intensity cytoplasmic expression **(A, A1)**, moderate-intensity cytoplasmic expression **(B, B1)**, high-intensity cytoplasmic expression **(C, C1)**, low-intensity membranous expression **(D, D1)**, moderate-intensity membranous expression **(E, E1)**, and high-intensity membranous expression **(F, F1)**. VISTA expression in adjacent non-tumoral tissue **(G)**. VISTA expression in kidney tissue as a positive control **(H)**. Isotype control **(I)**. Images were taken at 100 × **(A–F)** and 200× **(A1–F1)** magnification.

The protein expression levels of CD8, CD68, and VISTA were evaluated by IHC (**Figures 4**–**6**). CD68 and VISTA were expressed at different intensities in the cytoplasm and membrane (**Figures 5** and **6**), while CD8 was only expressed in the cytoplasm in both PDAC tissues and adjacent normal samples (**Figure 4**; **Table 2**). Although the median expression levels of CD8, CD68, and VISTA were higher in PDAC tissues compared to adjacent normal tissues, the Mann-Whitney U test indicated no statistically significant difference between the median cytoplasmic and membranous expression of VISTA, as well as membranous expression of CD68, and the median expression of normal tissues in terms of H-score (*P*=0.063, *P*=0.119, and *P*=0.321, respectively).

**Table 2:** Expression of VISTA, CD68, and CD8 in PDAC tumors.

Expression	Cytoplasmic expression			
	VISTA	CD68		CD8
Intensity of staining				
Negative (0)	35 (23.6)	24 (17.1)		67 (54.0)
Weak (+1)	64 (43.2)	22 (15.7)		15 (12.1)
Moderate (+2)	39 (26.4)	65 (46.4)		31 (25.0)
Strong (+3)	10 (6.8)	29 (20.7)		11 (8.9)
Percentage of positive tumor cells				
< 25%	124 (83.8)	100 (71.4)		106 (85.5)
25–50%	16 (10.8)	32 (22.9)		12 (9.7)
51–75%	0 (0.0)	5 (3.6)		2 (1.6)
> 75%	8 (5.4)	3 (2.1)		4 (3.2)
**H-score cut-off**	10	30		22
Low	85 (57.8)	79 (56.4)		78 (62.9)
High	63 (42.6)	61 (43.6)		46 (37.1)
Expression	Membranous expression			
	VISTA		CD68	
Intensity of staining				
Negative (0)	81 (54.7)		126 (90.0)	
Weak (+1)	36 (24.3)		8 (5.7)	
Moderate (+2)	19 (12.8)		6 (4.3)	
Strong (+3)	12 (8.1)		0 (0.0)	
Percentage of positive tumor cells				
< 25%	135 (91.2)		140 (100.0)	
25–50%	11 (7.4)		0 (0.0)	
51–75%	2 (1.4)		0 (0.0)	
> 75%	0 (0.0)		0 (0.0)	
**H-score cut-off**	16		2	
Low	116 (78.4)		126 (90.0)	
High	32 (21.6)		14 (10.0)	

The analysis showed a statistically significant difference between the median cytoplasmic CD68 and CD8 expression and the median expression of normal tissues in terms of H-score (*P*<0.001 and *P*=0.027, respectively). Positive controls using tonsil tissue for CD8 and CD68, and kidney tissue for VISTA were used, which showed strong staining in the cytoplasm and membrane.

### 3.5 Association between the expression of VISTA, CD68, and CD8 with clinicopathological features in PDAC samples

Pearson’s χ2 test was utilized to examine the association between VISTA and clinicopathological parameters. Our analysis indicated that the membranous expression of VISTA was associated with distant metastasis (*P*=0.002). However, there was no association between the cytoplasmic expression of VISTA and clinicopathological features (**Table 3**).

**Table 3:** The association between expression of VISTA and clinicopathological features of patients with pancreatic ductal adenocarcinoma carcinoma.

Tumor characteristics	Total samples N (%)	Cytoplasmic expression		*P-value*	Membranous expression		*P-value*
		H score (cut off = 10) N (%)			H score (cut off = 16) N (%)		
		Low (≤ 10)	High (> 10)		Low (≤ 16)	High (> 16)	
**Number of samples**	148	85	63		116	32	
**Median age, years (Range)** ≤ Median age > Median age	59 (12-85) 74 (50.0) 74 (50.0)	44 (51.8) 41 (48.2)	30 (47.6) 33 (52.4)	0.618	60 (51.7) 56 (48.3)	14 (43.8) 18 (56.3)	0.424
**Sex** Male Female	81 (54.7) 67 (45.3)	43 (50.6) 42 (49.4)	38 (60.3) 25 (39.7)	0.240	64 (55.2) 52 (44.8)	17 (53.1) 15 (46.9)	0.837
**Median tumor size (cm) (Range)** ≤ Median > Median	3 (0.3-16.5) 74 (51.0) 71 (49.0)	48 (57.1) 36 (42.9)	26 (42.6) 35 (57.4)	0.084	55 (48.7) 58 (51.3)	19 (59.4) 13 (40.6)	0.285
**Histological grade** Well-differentiated Moderate differentiated Poor differentiated	58 (44.9) 59 (45.7) 12 (9.4)	32 (43.8) 33 (45.2) 8 (11.0)	26 (46.4) 26 (46.4) 4 (7.2)	0.758	42 (41.6) 49 (48.5) 10 (9.9)	16 (57.1) 10 (35.7) 2 (7.2)	0.342
**TNM stage** I II III IV	29 (25.0) 68 (58.6) 15 (12.9) 4 (3.5)	21 (30.9) 37 (54.4) 9 (13.2) 1 (1.5)	8 (16.7) 31 (64.6) 6 (12.5) 3 (6.2)	0.200	21 (23.0) 55 (60.4) 12 (13.1) 3 (3.5)	8 (32.0) 13 (52.0) 3 (12.0) 1 (4.0)	0.822
**Margin involvement** Yes No	31 (25.2) 92 (74.8)	18 (25.7) 52 (74.3)	13 (24.5) 40 (75.5)	0.881	24 (25.3) 71 (74.7)	7 (25.0) 21 (75.0)	0.978
**Perineural invasion** Present Absent	83 (66.9) 41 (33.1)	49 (68.1) 23 (31.9)	34 (65.4) 18 (34.6)	0.755	67 (69.8) 29 (30.2)	16 (57.1) 12 (42.9)	0.211
**Lymphovascular invasion** Present Absent	61 (54.9) 50 (45.1)	37 (58.7) 26 (41.3)	24 (50.0) 24 (50.0)	0.360	45 (54.2) 38 (45.8)	16 (57.1) 12 (42.9)	0.788
**Lymph node (LN) metastasis** Present Absent	63 (52.9) 56 (47.1)	38 (54.3) 32 (45.7)	25 (51.0) 24 (49.0)	0.725	49 (52.1) 45 (47.9)	14 (56.0) 11 (44.0)	0.730
**Macroscopic tumor extension** Yes No	91 (71.0) 37 (29.0)	50 (70.4) 21 (29.6)	41 (71.9) 16 (28.1)	0.852	70 (70.7) 29 (29.3)	21 (72.4) 8 (27.6)	0.858
**Tumor recurrence** Yes No	13 (9.2) 128 (90.8)	9 (11.3) 71 (88.7)	4 (6.6) 57 (93.4)	0.340	11 (10.1) 98 (89.9)	2 (6.3) 30 (93.7)	0.509
**Distant metastasis** Yes No	60 (42.5) 81 (57.5)	37 (46.3) 43 (53.8)	23 (37.7) 38 (62.3)	0.309	54 (49.5) 55 (50.5)	6 (18.7) 26 (81.3)	0.002*

*P value; Pearson’s chi-square: The significance of the P value is determined using the Benjamini-Hochberg procedure, resulting in a value of 0.01.

H-score, Histological score.

Values in bold and italic are statistically signiﬁcant.

Subsequently, our analysis revealed a significant inverse correlation between the cytoplasmic expression of CD68 and lymphovascular invasion (*P*=0.002). Furthermore, it was discovered that there exists a significant positive correlation between membranous expression of CD68 and age (*P*=0.024; **Table 4**). Moreover, it was found that there exists no statistically significant association between cytoplasmic expression of CD8 and clinicopathological features (**Table 5**).

**Table 4:** The association between expression of CD68 and clinicopathological features of patients with pancreatic ductal adenocarcinoma carcinoma.

Tumor characteristics	Total samples N (%)	Cytoplasmic expression		*P value*	Membranous expression		*P value*
		H score (cut off = 30) N (%)			H score (cut off = 2) N (%)		
		Low (≤ 30)	High (> 30)		Low (≤ 2)	High (> 2)	
**Number of samples**	140	79	61		126	14	
**Median age, years (Range)** ≤ Median age > Median age	60 (19-85) 70 (50.0) 70 (50.0)	40 (50.6) 39 (49.4)	30 (49.2) 31 (50.8)	0.865	67 (53.2) 59 (46.8)	3 (21.4) 11 (78.6)	0.024
**Sex** Male Female	71 (50.7) 69 (49.3)	37 (46.8) 42 (53.2)	34 (55.7) 27 (44.3)	0.296	64 (50.8) 62 (49.2)	7 (50.0) 7 (50.0)	0.955
**Median tumor size (cm) (Range)** ≤ Median > Median	3 (0.4-10) 77 (55.7) 61 (44.3)	43 (55.8) 34 (44.2)	34 (55.7) 27 (44.3)	0.990	71 (57.3) 53 (42.7)	6 (42.9) 8 (57.1)	0.304
**Histological grade** Well-differentiated Moderate differentiated Poor differentiated	55 (44.3) 58 (46.7) 11 (9.0)	35 (52.2) 25 (37.3) 7 (10.5)	20 (35.1) 33 (57.9) 4 (7.0)	0.073	51 (45.5) 51 (45.5) 10 (9.0)	4 (33.3) 7 (58.3) 1 (8.4)	0.688
**TNM stage** I II III IV	45 (36.2) 58 (46.7) 14 (11.2) 7 (5.9)	25 (37.3) 31 (46.3) 7 (10.4) 4 (6.0)	20 (35.1) 27 (47.4) 7 (12.3) 3 (5.2)	0.982	40 (36.0) 51 (45.9) 13 (11.7) 7 (6.4)	5 (38.5) 7 (53.8) 1 (7.7) 0 (0.0)	0.762
**Margin involvement** Yes No	30 (23.4) 98 (76.6)	14 (20.3) 55 (79.7)	16 (27.1) 43 (72.9)	0.363	27 (23.7) 87 (76.3)	3 (21.4) 11 (78.6)	0.851
**Perineural invasion** Present Absent	77 (61.1) 49 (38.9)	38 (55.9) 30 (44.1)	39 (67.2) 19 (32.8)	0.192	71 (63.4) 41 (36.6)	6 (42.9) 8 (57.1)	0.137
**Lymphovascular invasion** Present Absent	56 (52.8) 50 (47.2)	22 (38.6) 35 (61.4)	34 (69.4) 15 (30.6)	0.002*	51 (54.8) 42 (45.2)	5 (38.5) 8 (61.5)	0.268
**Lymph node (LN) metastasis** Present Absent	64 (50.7) 62 (49.3)	33 (47.8) 36 (52.2)	31 (54.4) 26 (45.6)	0.464	59 (52.2) 54 (47.8)	5 (38.5) 8 (61.5)	0.348
**Macroscopic tumor extension** Yes No	94 (77.0) 28 (23.0)	45 (70.3) 19 (29.7)	49 (84.5) 9 (15.5)	0.063	85 (78.7) 23 (21.3)	9 (64.3) 5 (35.7)	0.227
**Tumor recurrence** Yes No	20 (14.7) 116 (85.3)	8 (10.5) 68 (89.5)	12 (20.0) 48 (80.0)	0.121	19 (15.6) 103 (84.4)	1 (7.1) 13 (92.9)	0.399
**Distant metastasis** Yes No	59 (43.3) 77 (56.7)	30 (39.5) 46 (60.5)	29 (48.3) 31 (51.7)	0.301	53 (43.4) 69 (56.6)	6 (42.9) 8 (57.1)	0.967

*P value; Pearson’s chi-square: The significance of the P value is determined using the Benjamini-Hochberg Procedure, resulting in a value of 0.01.

H-score, Histological score.

Values in bold and italic are statistically signiﬁcant.

**Table 5:** The association between cytoplasmic expression of CD8 and clinicopathological features of patients with pancreatic ductal adenocarcinoma carcinoma.

Tumor characteristics	Total samples N (%)	Cytoplasmic expression		*P value*
		H score (cut off = 22) N (%)		
		Low (≤ 22)	High (> 22)	
Number of samples	124	78	46	
Age				
**Median Age, years (Range)** ≤ Median age > Median age	59 (12-85) 65 (52.4) 59 (47.6)	42 (53.8) 36 (46.2)	23 (50.0) 23 (50.0)	0.679
Sex				
Male Female	60 (48.4) 64 (51.6)	37 (47.4) 41 (52.6)	23 (50.0) 23 (50.0)	0.783
Tumor size				
**Median Tumor size (cm) (Range)** ≤ Median > Median	3.3 (0.5-16.5) 61 (50.0) 61 (50.0)	37 (48.1) 40 (51.9)	24 (53.3) 21 (46.7)	0.573
Histological grade				
Well-differentiated Moderate differentiated Poor differentiated	52 (45.2) 55 (47.8) 8 (7.0)	30 (40.5) 38 (51.4) 6 (8.1)	22 (53.7) 17 (41.5) 2 (4.9)	0.379
TNM stage				
I II III IV	33 (31.4) 55 (52.3) 13 (12.3) 4 (4.0)	20 (30.8) 35 (53.8) 8 (12.3) 2 (3.1)	13 (32.5) 20 (50.0) 5 (12.5) 2 (5.0)	0.953
Margin involvement				
Yes No	30 (27.2) 80 (72.8)	21 (30.4) 48 (69.6)	9 (22.0) 32 (78.0)	0.334
Perineural invasion				
Present Absent	77 (71.2) 31 (28.8)	48 (71.6) 19 (28.4)	29 (70.7) 12 (29.3)	0.919
Lymphovascular invasion				
Present Absent	52 (52.5) 47 (47.5)	33 (55.0) 27 (45.0)	19 (48.7) 20 (51.3)	0.541
Lymph node (LN) metastasis				
Present Absent	55 (51.8) 51 (48.2)	35 (53.0) 31 (47.0)	20 (50.0) 20 (50.0)	0.762
Macroscopic tumor extension				
Yes No	83 (74.7) 28 (25.3)	54 (78.3) 15 (21.7)	29 (69.0) 13 (31.0)	0.278
Tumor recurrence				
Yes No	12 (10.0) 107 (90.0)	8 (10.7) 67 (89.3)	4 (9.1) 40 (90.9)	0.783
Distant metastasis				
Yes No	57 (47.8) 62 (52.2)	39 (52.0) 36 (48.0)	18 (40.9) 26 (59.1)	0.242

P value; Pearson’s chi-square: The significance of the P value is determined using the Benjamini-Hochberg procedure, resulting in a value of 0.01.

H-score; Histological score.

### 3.6 Co-expression of VISTA/CD68/CD8 markers with clinicopathological parameters in PDAC

The results demonstrated a statistically significant direct correlation between cytoplasmic expression of VISTA and CD8 expression (*P*=0.028). However, there was no correlation between VISTA and CD68, or between CD68 and CD8. Furthermore, the association between the co-expression of VISTA/CD8 proteins with clinicopathological features was examined through Pearson’s χ2 square test. The expression levels of VISTA and CD8 were divided into two categories based on median expression: low and high expression. Therefore, there were four phenotypes comprising VISTA^High^/CD8^High^, VISTA^High^/CD8^Low^, VISTA^Low^/CD8^High^, and VISTA^Low^/CD8^Low^. The statistical analysis revealed no significant association between the co-expression of VISTA/CD8 and clinicopathological sample characteristics.

### 3.7 Survival analysis based on VISTA expression

**Figure 7:**
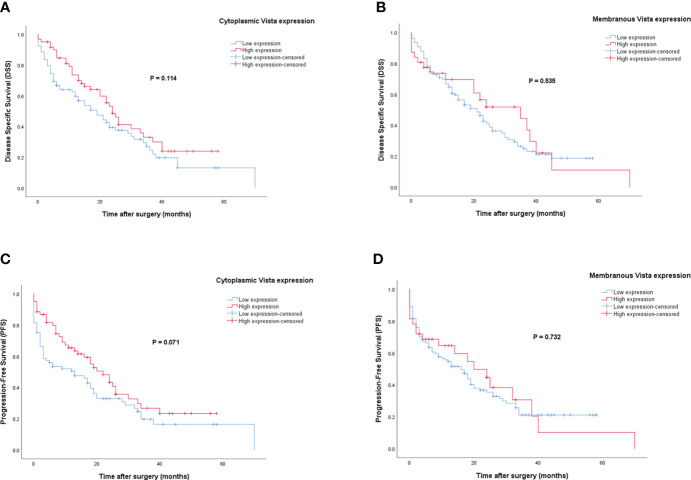
Kaplan-Meier survival curves for disease-specific survival (DSS) and progression-free survival (PFS) based on cytoplasmic and membranous VISTA protein expression levels in pancreatic ductal adenocarcinoma (PDAC). The Kaplan-Meier survival analysis showed no significant differences between DSS or PFS and the patients with high and low cytoplasmic **(A, C)**, and membranous **(B, D)** expression of VISTA protein.

Patients with VISTA expression had a median DSS and PFS of 15 (Q1, Q3: 6, 28) and 12 months (Q1, Q3: 2, 24), respectively. The mean DSS and PFS follow-up time for patients with high cytoplasmic and membranous expression of VISTA was longer compared to those with low expression. However, Kaplan-Meier survival analysis indicated that there were no statistically significant differences between DSS (Log-rank test; cytoplasmic: *P*=0.114 and membranous: *P*=0.535) or PFS (Log-rank test; cytoplasmic: *P*=0.071 and membranous: *P*=0.732) and the patients with high/low expression of VISTA (**Figure 7**).

### 3.8 Survival analysis based on CD68 expression

**Figure 8:**
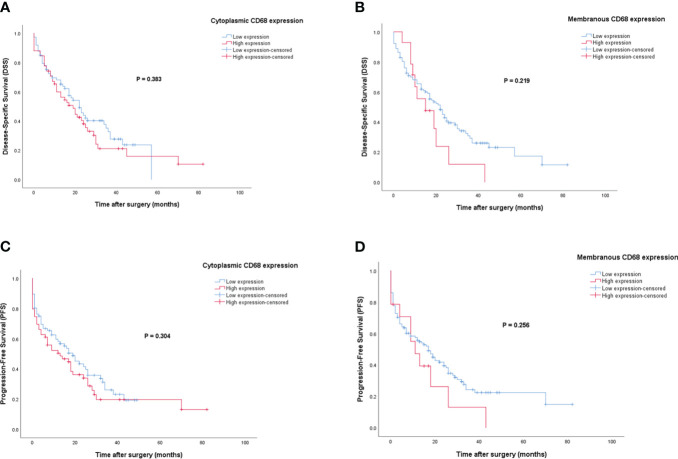
Kaplan-Meier survival curves for disease-specific survival (DSS) and progression-free survival (PFS) based on cytoplasmic and membranous CD68 protein expression levels in pancreatic ductal adenocarcinoma (PDAC). The Kaplan-Meier survival analysis showed no significant differences between DSS or PFS and the patients with high and low cytoplasmic **(A, C)**, and membranous **(B, D)** expression of CD68 protein.

For patients with CD68 expression, the median DSS and PFS were 17 (Q1, Q3: 6, 27) and 12 months (Q1, Q3: 2, 26), respectively. In contrast to VISTA, patients with high cytoplasmic and membranous expression of CD68 have shorter DSS and PFS than those with low expression. However, Kaplan-Meier survival analysis showed no significant differences between DSS (Log-rank test; cytoplasmic: *P*=0.383 and membranous: *P*=0.219) or PFS (Log-rank test; cytoplasmic: *P*=0.304 and membranous: *P*=0.256) and the patients with high/low expression of CD68 (**Figure 8**).

### 3.9 Survival analysis based on CD8 expression

**Figure 9:**
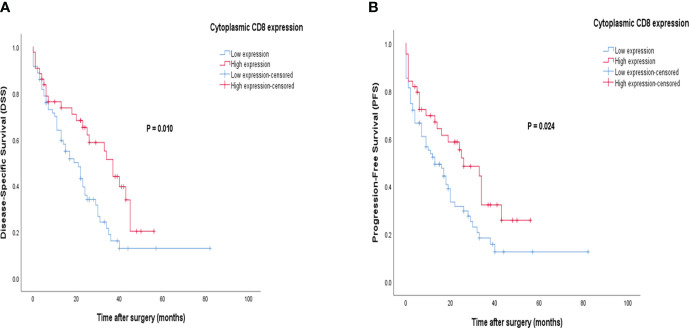
Kaplan-Meier survival curves for disease-specific survival (DSS) and progression-free survival (PFS) based on cytoplasmic CD8 protein expression levels in pancreatic ductal adenocarcinoma (PDAC). The Kaplan-Meier survival analysis showed significant differences between DSS or PFS and the patients with high and low cytoplasmic **(A, B)** expression of CD8 protein.

Patients with CD8 expression showed a median DSS and PFS of 17 months (Q1 and Q3: 6 and 30 months) and 13 months (Q1 and Q3: 3 and 26 months), respectively. Patients with a high cytoplasmic expression of CD8 had longer DSS and PFS follow-up times than those with a low expression. The results of the Kaplan-Meier curve confirmed significant differences between DSS (Log-rank test; *P*=0.010) and PFS (Log-rank test; *P*=0.024) and the patients with high/low expression of CD8 (**Figure 9**), indicating that patients with high CD8 expression had significantly better survival. Besides, univariate and multivariate Cox regression analysis were conducted to determine the clinical significance of various parameters that may have influenced DSS and PFS. In the univariate analysis, cytoplasmic expression of CD8, tumor size, grade, and metastasis were significant risk factors affecting the DSS and PFS of patients. Then, the parameters that impacted survival in the univariate analysis were included in the multivariable Cox proportional hazards regression analysis. As shown in **Table 6**, tumor size, grade, and metastasis were independent prognostic factors for DSS and PFS in the multivariate analysis.

**Table 6:** Univariate and multivariate cox regression analysis of potential prognostic factors for Disease-Specific (DSS) and Progression-Free Survival (PFS) in patients with pancreatic ductal adenocarcinoma.

Covariate	Disease-Specific Survival (DSS)				Progression-Free Survival (PFS)			
	Univariate analysis		Multivariate analysis		Univariate analysis		Multivariate analysis	
	HR (95% CI)	*P-value*	HR (95% CI)	*P-value*	HR (95% CI)	*P-value*	HR (95% CI)	*P-value*
**High cytoplasmic CD8 expression versus low expression**	0.532 (0.324-0.875)	***0.013***	0.643 (0.368-1.123)	0.120	0.588 (0.365-0.948)	***0.029***	0.692 (0.405-1.180)	0.176
**Median age**	1.471 (0.935-2.315)	0.095	–	–	1.446 (0.932-2.244)	0.100	–	–
**Gender**	0.932 (0.592-1.467)	0.762	–	**-**	1.004 (0.647-1.558)	0.986	–	–
**Median tumor size**	1.627 (1.025-2.582)	***0.039***	1.721 (1.042-2.843)	***0.034***	1.734 (1.111-2.707)	***0.015***	1.866 (1.153-3.019)	***0.011***
**Histological grade** **II versus I** **III versus I**	0.409 (0.167-1.001) 0.773 (0.324-1.844)	***0.024*** ***0.050*** 0.561	0.328 (0.131-0.822) 0.523 (0.214-1.281)	***0.035*** ***0.017*** 0.156	0.516 (0.213-1.252) 0.998 (0.421-2.364)	***0.026*** 0.144 0.996	0.395 (0.159-0.982) 0.720 (0.300-1.730)	***0.030*** ***0.046*** 0.463
**Stage** **II versus I** **III versus I** **IV versus I**	0.426 (0.123-1.481) 0.669 (0.203-2.204) 0.939 (0.257-3.437)	0.194 0.180 0.508 0.925	–	**-**	0.494 (0.144-1.692) 0.728 (0.222-2.382) 0.990 (0.272-3.608)	0.291 0.262 0.599 0.988	–	–
**Margin involvement**	0.635 (0.383-1.053)	0.078	–	**-**	0.555 (0.338-0.910)	0.060	–	–
**Perineural invasion**	0.817 (0.469-1.422)	0.474	–	**-**	0.789 (0.465-1.338)	0.379	–	–
**Lymphovascular invasion**	0.674 (0.392-1.160)	0.154	–	**-**	0.782 (0.467-1.309)	0.349	–	–
**Lymph node (LN) metastasis**	0.615 (0.372-1.014)	0.056	–	**-**	0.670 (0.413-1.086)	0.104	–	–
**Macroscopic tumor extension**	1.051 (0.581-1.901)	0.869	–	**-**	1.026 (0.593-1.774)	0.928	–	–
**Tumor recurrence**	1.069 (0.513-2.228)	0.858	–	**-**	0.861 (0.430-1.726)	0.674	–	–
**Distant metastasis**	0.458 (0.285-0.737)	***0.001***	0.453 (0.272-0.756)	***0.002***	0.421 (0.266-0.666)	***<0.001***	0.388 (0.235-0.641)	***<0.001***

HR, hazard ratio; CI, Confidence interval.

The variables with P value less than 0.05 were included in multivariable analysis.

Values in bold and italic are statistically significant.

## 4 Discussion

PDAC is the most prevalent form of pancreatic cancer, accounts for about 90% of all cases, and arises from the pancreatic epithelial cells. The aggressive behavior of PDACs is attributed to their rapid infiltration and growth patterns (35). Despite advances in treatment options, the prognosis for PDAC remains poor due to its advanced stage at diagnosis and resistance to current therapies (36). Thus, it is of paramount importance to discover novel functional indicators for prognosis and diagnosis and develop novel therapeutic techniques.

In the current study, CD8, CD68, and VISTA molecules were selected as markers for evaluation in pancreatic cancer due to their well-known roles in regulating immune responses, which are of particular importance in cancer progression. CD8 is expressed on the surface of cytotoxic T cells as a co-receptor in association with the T cell receptor, making them the strongest effectors in fighting against cancer through the immune system and serving as a foundation for effective cancer immunotherapies (37). CD68 is a commonly used marker to detect M2-like TAMs in the TME, which have been shown to promote cancer growth and metastasis through various mechanisms, including immune suppression and angiogenesis (38, 39). In addition, CD68 has also been identified as a marker for M1 macrophages, which have anti-tumor functions and are associated with a favorable prognosis in some cancers (40, 41). VISTA, also known as PD-1H, Gi24, Dies-1, and DD1α, is an inhibitory immune checkpoint protein belonging to the B7 family that has the potential to regulate the immune response of both myeloid and lymphoid lineages (42, 43).

Based on *in silico* data, particularly the STRING PPI network, interactions exist between the CD8A, CD68, and VISTA genes and other important genes such as B2M, CD4, CD80, IL2, CTLA-4, CD86, CD28, and PTPRC. The results demonstrated that a robust correlation exists between VISTA and CTLA-4, which suggests its potential role in cancer development and progression. CTLA-4, also referred to as CD152, is a transmembrane protein that has a close association with CD28 despite playing distinct roles in the immune response. CD28, which is a costimulatory receptor, is located on both CD4^+^ and CD8^+^ T cell surfaces and activates the entire cell by sending a signal when it interacts with CD80 (B7-1) dimer and CD86 (B7-2) monomer ligands, in addition to the signal from TCR. Unlike CD28, CTLA-4 is mainly found in intracellular vesicles and has a higher affinity for CD80 and CD86. It competes with CD28 for binding ligands and subsequently forms the CTLA-4-CD80 complex or the CTLA-4-CD86 complex, which is transported to the cytoplasm and eliminated by lysosomal compartments. This process eventually suppresses the activation of T cells (44–46). VISTA (B7-H5) demonstrates its homology with CTLA-4 and CD28 (47). Further investigations are required to elucidate the precise relationship between VISTA and CTLA-4. However, despite limited data, a study conducted by Kondo et al. suggested that combining anti-CTLA-4 and anti-VISTA therapies may yield superior therapeutic outcomes (48). According to a study by Gao et al., after administering ipilimumab (anti-CTLA-4) therapy to patients with prostate cancer, the presence of VISTA and PD-L1 inhibitory molecules was observed to increase within the macrophages in the treated tumors (49). The investigation of this network also uncovered that CD8A, CD68, and VISTA are subject to substantial influence by essential proteins and transcription factors, including TP53, EP300, ESR1, CCND1, CREBBP, FOS, HDAC1, SMAD3, SMAD2, and STAT1, which are associated with cancer signaling pathways, indicating their involvement in the development of cancer. For example, Schlichtner et al. reported that the transforming growth factor beta type 1 (TGF-β)-Smad3 signaling pathway is responsible for regulating the expression of VISTA (50). Wang et al. revealed that the maintenance of the CD68^+^ TAMs phenotype was mediated by miR-100 through the involvement of the mTOR pathway, indicating the diverse functions of the mTOR pathway in regulating the macrophage phenotype network (51).

The analysis of the data from two different sources, including GEO, and an IHC experiment, revealed some interesting findings regarding the expression of three markers in PDAC. The first marker, VISTA, according to the bioinformatics data, the augmentation in the level of mRNA expression is not remarkable, albeit mild. However, IHC indicates a noteworthy elevation in its expression in PDAC. VISTA is overexpressed in several malignancies, like breast cancer (52), hepatocellular carcinoma (HCC) (53), gastric cancer (54), colorectal cancer (55), gliomas (56), brain metastasis of lung adenocarcinoma (57), non-small cell lung cancer (NSCLC) (58), oral squamous cell carcinoma (59), cervical cancer (60), ovarian cancer (61), endometrial cancer (32), clear cell renal cell carcinoma (62), and gestational trophoblastic neoplasia (63). However, some studies have shown that VISTA can be downregulated in different tumors (47, 64). VISTA is known to have an inhibitory effect on T cell activation, which could explain the high expression levels observed in this study. VISTA expression is particularly high on certain types of T cells, specifically naïve CD4^+^ T cells, and regulatory T cells expressing Foxp3 (47). Studies have indicated that VISTA is vital for the quiescence of naïve CD4^+^ T cells. If VISTA is absent or blocked, it decreases the activation threshold of T cells, leading to elevated T cell responses to self-antigens (65). Recent studies have demonstrated that anti-tumor immunity, particularly T cell-mediated immunity, and the effectiveness of responses to tumor-associated antigens may be promoted by either genetic VISTA deficiency or the use of an anti-VISTA monoclonal antibody antagonist (66, 67). Also, Schaafsma et al. demonstrated that in the CT26 colorectal cancer model, the addition of anti-VISTA to anti-CTLA-4/anti-PD-1 treatment reduced the expression of genes controlling quiescence, resulting in increased mature cytotoxic CD8^+^ T-cell subsets, demonstrating that VISTA plays a significant role in maintaining T-cell quiescence (66). Hong et al. showed that the expression of VISTA had a strong correlation with a decrease in CD8^+^ T cell responses and inhibiting VISTA signaling resulted in a significant reduction in the growth of the mouse RCC model. Another study by them demonstrated that the administration of anti-VISTA treatment led to a significant increase in the percentage of granzyme B^+^ Perforin^+^ CD8^+^ T cells in most patients with clear cell renal cell carcinoma (62, 68). Moreover, when it comes to myeloid lineages, VISTA has been shown to have immunosuppressive roles, and the use of anti-VISTA treatment has been found to significantly alter the function of myeloid cells within tumors. For instance, Le Mercier et al. found that blocking VISTA had an impact on the immune-suppressive nature of the TME of mouse melanoma cell lines by reducing the abundance of monocytic MDSCs and increasing the abundance of activated dendritic cells within the TME (69). Anti-VISTA treatment induces a shift from a suppressive phenotype to an activated state in colorectal cancer by increasing the expression of genes related to antigen presentation and interferon-regulated pathways (66). VISTA deficiency also disrupts myeloid cell chemotaxis and increases the accumulation of chemokines and inflammatory cytokines (70). Nonetheless, there were discrepancies in the expression levels of the other two markers, CD8 and CD68, between the two sources. IHC analysis revealed significantly high cytoplasmic expression of CD8 and CD68 in PDAC compared to adjacent normal tissue, while according to bioinformatics approaches, when it comes to CD8, GEO showed low expression levels. On the other hand, GEO showed high levels of CD68 expression as indicated by IHC. These differences across the two sources may be due to various factors, such as differences in sample size and sample preparation methods, and they highlight the complexity and heterogeneity of the TME and underscore the importance of using multiple approaches to understand the immune landscape of tumors. The expression of CD68 is elevated in assorted types of cancer (41, 71, 72). Macrophages that exhibit M2-like characteristics, like CD68^+^ TAMs, release a variety of cytokines and chemokines with anti-inflammatory properties, which have been shown to support the growth and spread of tumors (41, 73). CD68^+^ TAMs promote tumor growth and angiogenesis (74). Despite this, in some investigations, it has been shown that the expression of CD68 is lower in tumoral tissues (41). Thus, the role of CD68 in cancer is intricate and context-dependent, and further studies are needed to fully understand its function in assorted types of cancer.

Co-expression analysis revealed a positive correlation between the cytoplasmic expression of VISTA and CD8. This suggests that VISTA and CD8 may have a functional relationship in the TME. This finding is consistent with previous studies that have shown a correlation between VISTA and CD8 expression in various types of cancer, like HCC (75), ovarian cancer (61, 76), NSCLC (58), and triple-negative breast cancer (TNBC) (64). Furthermore, in solid tumors, He et al. reported a significant association between high VISTA expression and increased numbers of CD8^+^ tumor-infiltrating lymphocytes (TILs) (77). In addition, while our study did not show a co-expression of CD68 and VISTA, several other studies have reported such co-expression in various types of cancer, particularly in PDAC (28, 30, 58, 78, 79). In a study conducted by Hou et al., it was discovered through multiplex immunofluorescence analysis that there was a positive correlation between VISTA levels and CD68^+^ TAMs in pancreatic cancer. Besides, VISTA expression was found to be notably higher in CD68^+^ TAMs compared to CD3^+^ TILs or CD19^+^ B cells (78). Blando et al. also reported that in PDACs, VISTA expression is mainly observed on CD68^+^ macrophages (30). The discrepancies may have a linkage to several factors including different experimental techniques, specific microenvironment conditions, and other aspects such as patient heterogeneity, sample size, and study design.

Our results revealed that there was a significant association between high membranous expression of VISTA and distant metastasis. Furthermore, there was a notable negative correlation between CD68 cytoplasmic expression and lymphovascular invasion, indicating that tumors with less lymphovascular invasion displayed a higher level of CD68 expression. Furthermore, we observed a significant positive correlation between age and CD68 membranous expression. However, there was no statistically significant association between CD8 cytoplasmic expression and clinicopathological features. Lu et al. investigated the relationship between CD8^+^ TILs and CD68^+^ TAMs with clinicopathological characteristics in gastric cancer. They discovered that a positive CD8^+^ tumor-infiltrating status was inversely associated with lymphovascular invasion, while there was no significant correlation between CD68^+^ TAMs and clinicopathological features (80). Metastatic PDACs exhibit lower levels of total T cell infiltration (CD3, CD4, and CD8) when compared to resectable primary PDACs (30). Popp et al. found no association between VISTA and clinicopathological features (81). Patients with N0 stage, T1-2 stage, low tumor grade, and high CD8 density show higher VISTA expression on immune cells in colorectal cancer (82). Immune cells expressing VISTA in the TME of TNBC show a correlation with the absence of metastasis in lymph nodes (83). A positive correlation is observed between VISTA expression in the immune cells of patients with bladder cancer, including non-muscle invasive bladder cancer, and clinicopathological features such as tumor grade, stage, size, and multiplicity (84).

According to our IHC data, the expression levels of CD68 and VISTA did not demonstrate any significant association with patient survival. Nonetheless, an increase in CD8 levels was found to be associated with a higher probability of survival. This may be because CD8^+^ T cells are crucial in the anti-tumor immune response as they recognize and eliminate tumor cells (85). In addition, several studies have confirmed the association between high CD8^+^ TILs and better survival in various malignancies (86–89). Masugi et al. found that CD8^+^ T cell infiltration in the tumor center of pancreatic cancer was limited, and higher densities of these cells were associated with prolonged patient survival (90). Popp et al. revealed that there was no association between VISTA expression in PDAC and survival parameters (81). Hou et al. reported that although VISTA expression in immune cells and endothelial cells did not show an association with patient survival, there was a significant correlation between high levels of VISTA expression in pancreatic tumor cells and improved overall survival (OS) (78). Better OS is associated with high expression of PD-L1 or VISTA on immune cells present in the TME of PDAC (91). In contrast, in a study by Blando et al., survival was found to have an inverse correlation with the expression of the VISTA gene (30). Similarly, Loch et al. demonstrated that the existence of VISTA alone and in combination with PD-L1 was strongly linked to reduce OS in PDAC. Furthermore, their findings indicated that the expression of VISTA was not uniform across tumors and only exerted an influence on OS when it was detected in the central region of the tumor (92). One reason for the controversy surrounding VISTA’s association with survival could be methodological differences between these studies. Regarding other cancers, VISTA expression has been shown to have conflicting outcomes in terms of survival. Several studies have shown that VISTA expression is associated with immune suppression and poorer survival in different types of malignancies like melanoma (93, 94), gliomas (56), NSCLC (95), and human papillomavirus (HPV)-infected cervical cancer (96). On the other hand, some studies have shown that the presence of VISTA on the tumor may indicate an ongoing immune response against the tumor, which could cause better survival and serve as a favorable prognostic factor, like for oral squamous cell carcinoma (97) and NSCLC (58). In support of this, the presence of both VISTA and CD8^+^ markers is linked to a favorable TME and improved OS, as seen in HCC (75), esophageal adenocarcinoma (79), ovarian cancer (61), and TNBC (64). In addition, a meta-analysis of various types of solid tumors highlighted that there was a positive correlation between high levels of VISTA expression in tumors, increased T cell infiltration, and improved OS (77). In terms of CD68, many studies have reported its association with lower survival and a poor prognosis. In PDAC, a strong association was observed by Diana et al. between high CD68 expression in the tumor compartment and poorer PFS and distant metastasis-free survival (98). Zhang et al. demonstrated that elevated CD68 levels in tumor specimens were found to be associated with an unfavorable prognosis in various cancers, including glioblastoma, lower-grade glioma, clear-cell renal carcinoma, HCC, squamous cell carcinoma of the lung, thyroid carcinoma, and thymoma. However, in kidney chromophobe, higher CD68 expression was linked with a better prognosis (41). In contrast, some studies have indicated that low CD68 levels are associated with shorter survival, like in papillary thyroid cancer (99).

To date, a large number of tumor markers have been discovered and studied for their diagnostic value in pancreatic cancer. These include carbohydrate antigens (CA19-9, CA125, CA50, CA242), glycoproteins (CEA, POA), and non-coding RNAs (100–102). Among these, CA19-9 has been the most extensively validated biomarker (102). Due to ROC cure analysis, as there were no false positive results observed, all markers displayed high specificity but low sensitivity. This suggests that VISTA expression (both cytoplasmic and membranous), as well as cytoplasmic expression of CD68 and CD8, had significant diagnostic value and could serve as potential markers for PDAC diagnosis. However, further investigations are needed to confirm the diagnostic value of these markers and to determine their clinical usefulness in pancreatic cancer diagnosis.

Based on the results of our study, we found that VISTA, CD8, and CD68 expression levels were significantly associated with clinicopathological features and survival outcomes in PDAC. Our findings suggest that these markers may have diagnostic value and potential as therapeutic targets for cancer treatment. Immune checkpoint blockade has proven to be an effective treatment for various advanced cancers. VISTA monoclonal antibodies had a considerable inhibitory effect on the growth of melanoma tumors (69) and ovarian tumors (32) in mice. A previous study also showed that combining anti-VISTA and anti-PD-L1 antibodies had a synergistic therapeutic effect in a mouse model of colon cancer (29). By targeting VISTA expressed in immune cells, anti-VISTA therapy has the potential to disrupt the immune escape process and suppress tumor growth (78). Overall, our data support the notion of VISTA as an important player in PDAC and as a potential immunotherapeutic target.

The current immunotherapy used for PDAC has not achieved the desired level of success compared to its effectiveness in treating other types of solid tumors. As a result, additional therapeutic methods are required. VISTA has been identified as a powerful inhibitory checkpoint on CD68^+^ macrophages when comparing a tumor that responds well to immunotherapy with a tumor that does not respond well to immunotherapy (30). VISTA serves as a compensatory inhibitory pathway in cases of prostate cancer following ipilimumab therapy, potentially leading to unsuccessful treatment outcomes (49). Colorectal cancer also demonstrated comparable results, as anti-VISTA therapy proved effective in conquering resistance to anti-PD-1/CTLA-4 treatment (66). Similar to a recent study (103), our data confirm that infiltrating immune cells highly express membranous VISTA in human PDAC.

Our study had several limitations, including the retrospective nature of the analysis, the relatively small sample size, and the lack of functional validation of these markers. Therefore, further studies are needed to fully understand the immunological aspects of the PDAC microenvironment and the potential role of these markers in shaping the immune response, validate our findings, and investigate the underlying mechanisms by which these markers contribute to cancer progression and treatment response.

## 5 Conclusion

The present study underscores the potential of CD8, CD68, and VISTA as diagnostic and prognostic indicators in PDAC. These results shed light on the functions of these markers in the progression and prognosis of PDAC, indicating their usefulness in the creation of more precise diagnostic instruments and targeted treatments for pancreatic cancer. Additional investigation is necessary to confirm these results and examine their therapeutic implications.

## Data availability statement

The datasets presented in this study can be found in online repositories. The names of the repository/repositories and accession number(s) can be found in the article/supplementary material.

## Ethics statement

The studies involving humans was conducted with ethical approval (Code: IR.IUMS.FMD.REC.1399.161) obtained from the Research Ethics Committee of the Iran University of Medical Sciences. The studies were conducted in accordance with the local legislation and institutional requirements. Written informed consent for participation in this study was provided by the participants’ legal guardians/next of kin.

## Author contributions

FR: Conceptualization, Data curation, Formal analysis, Investigation, Methodology, Project administration, Validation, Visualization, Writing – original draft, Writing – review & editing. FT: Data curation, Formal analysis, Methodology, Writing – original draft. MT: Data curation, Formal analysis, Validation, Visualization, Writing – review & editing, Investigation, Software, Writing – original draft. ST: Data curation, Formal analysis, Investigation, Writing – original draft. MS: Writing – original draft, Data curation, Formal analysis, Investigation. PF: Data curation, Formal analysis, Investigation, Validation, Visualization, Writing – review & editing, Writing – original draft. HH: Investigation, Writing – review & editing, Data curation, Formal analysis. AN: Data curation, Investigation, Writing – review & editing, Formal analysis. SM: Data curation, Investigation, Writing – review & editing, Formal analysis. SK: Data curation, Investigation, Resources, Writing – review & editing, Formal analysis. NH: Data curation, Investigation, Resources, Writing – review & editing, Formal analysis. HK: Data curation, Formal analysis, Writing – review & editing, Software, Visualization. MJ: Conceptualization, Data curation, Formal analysis, Funding acquisition, Investigation, Methodology, Project administration, Resources, Supervision, Validation, Writing – review & editing. ES: Conceptualization, Data curation, Formal analysis, Funding acquisition, Investigation, Methodology, Project administration, Resources, Supervision, Validation, Writing – review & editing.
